# RNA-seq Profiles of Immune Related Genes in the Staghorn Coral *Acropora cervicornis* Infected with White Band Disease

**DOI:** 10.1371/journal.pone.0081821

**Published:** 2013-11-21

**Authors:** Silvia Libro, Stefan T. Kaluziak, Steven V. Vollmer

**Affiliations:** Marine Science Center, Northeastern University, Nahant, Massachusetts, United States of America; Uppsala University, Sweden

## Abstract

Coral diseases are among the most serious threats to coral reefs worldwide, yet most coral diseases remain poorly understood. How the coral host responds to pathogen infection is an area where very little is known. Here we used next-generation RNA-sequencing (RNA-seq) to produce a transcriptome-wide profile of the immune response of the Staghorn coral *Acropora cervicornis* to White Band Disease (WBD) by comparing infected versus healthy (asymptomatic) coral tissues. The transcriptome of *A. cervicornis* was assembled de novo from A-tail selected Illumina mRNA-seq data from whole coral tissues, and parsed bioinformatically into coral and non-coral transcripts using existing *Acropora* genomes in order to identify putative coral transcripts. Differentially expressed transcripts were identified in the coral and non-coral datasets to identify genes that were up- and down-regulated due to disease infection. RNA-seq analyses indicate that infected corals exhibited significant changes in gene expression across 4% (1,805 out of 47,748 transcripts) of the coral transcriptome. The primary response to infection included transcripts involved in macrophage-mediated pathogen recognition and ROS production, two hallmarks of phagocytosis, as well as key mediators of apoptosis and calcium homeostasis. The strong up-regulation of the enzyme allene oxide synthase-lipoxygenase suggests a key role of the allene oxide pathway in coral immunity. Interestingly, none of the three primary innate immune pathways - Toll-like receptors (TLR), Complement, and prophenoloxydase pathways, were strongly associated with the response of *A. cervicornis* to infection. Five-hundred and fifty differentially expressed non-coral transcripts were classified as metazoan (n = 84), algal or plant (n = 52), fungi (n = 24) and protozoans (n = 13). None of the 52 putative *Symbiodinium* or algal transcript had any clear immune functions indicating that the immune response is driven by the coral host, and not its algal symbionts.

## Introduction

The global rise in disease epidemics linked to climate change has taken a heavy toll on tropical reef-building corals and the diverse ecosystems they support [1-4]. A prime example is White Band Disease (WBD), which beginning in the late 1970s [5], caused unprecedented Caribbean-wide die-offs of two species of *Acropora* corals, the Staghorn coral *A. cervicornis* and the Elkhorn coral *A. palmata* [6-8]. As a result, both species are now listed as threatened on the US Endangered Species Act [9] and as critically endangered under the International Union for the Conservation of Nature (IUCN) Red List criteria [4]. Despite the devastating impacts of coral diseases on reefs world-wide, little is known about the basic etiology and ecology of most coral diseases [10-12] including basic information about how corals fight diseases [2,12,13], even though information about the coral immune response may be crucial to understanding the future resiliency of reef corals [2].

Genetic surveys indicate that corals and other cnidarians possess the genetic architecture underlying common innate immune pathways, including Toll-like receptors (TLR) as well as components of the complement and prophenoloxidase (PO) pathways [10,14,15]. PO activity and melanization responses have been elicited in corals exposed to pathogens [16-18] and components of the TLR pathway were differentially expressed in corals infected with non-host specific *Symbiodinium* types [19]. Elements of the complement pathway, such as mannose-binding lectins, appear to be involved in pathogen, symbiont, and self/nonself recognition in *Acropora millepora* [20]. Although cnidaria lack specialized immune cells, such as macrophages, cnidaria possess mobile amebocytes that are activated upon pathogen exposure or tissue damage [21-24]. Phagocytosis activity in cnidarians is commonly observed in flagellate gastrodermal cells during food uptake [25]. However, several studies have demonstrated that, upon immune stimulation, different populations of amebocytes can exhibit phagocitic activity directed toward wound healing and removal of necrotic tissue, as well as encapsulation of foreign particles [26,27].

Relatively few studies have studied the genetic response of corals infected with disease [28,29]. A microarray study of *Pocillopora damicornis* infected with *Vibrio* identified six candidate immune genes including three lectins and three putative antimicrobial proteins [28]. Exposure of *A. millepora* to bacterial and viral pathogen associated molecular patterns (PAMPs) resulted in up-regulation of few immune related genes including three GTPase of immunity associated proteins (GiMAP) [29], a family of conserved small GTPases involved in the antibacterial response of plants and mammals [30].

**Figure 1:**
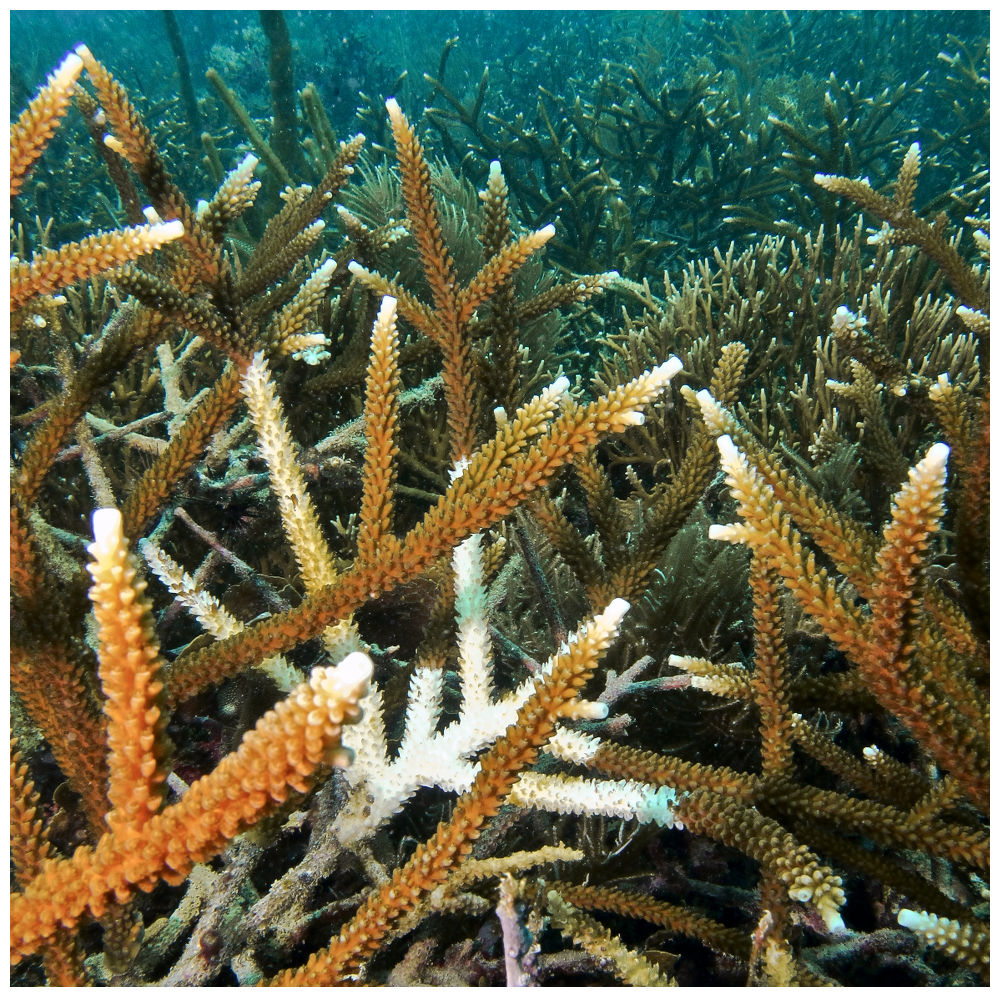
White Band Disease on *Acropora cervicornis*. A colony of the Staghorn coral *A. cervicornis* infected with White Band Disease showing the characteristic white band of dying and necrotic coral tissue.

White Band Disease represents a good system to investigate the immune response of a reef-building coral. It is one of the few coral diseases that is highly transmissible [31] and host-specific [5,11]. WBD is characterized by an interface of white dying tissue that advances rapidly along the coral colony (Figure 1). Current evidence suggests that the pathogen is bacterial [31-36], but Henle-Koch postulates have not been satisfied. To date, multiple bacteria have been associated with WBD infections, including *Vibrio harveyi* [33,37] as well as a marine *Rickettsia* CAR1α [34]. *In situ* transmission experiments have identified naturally resistant and susceptible genotypes of *A. cervicornis* [31], indicating that the immune response to WBD varies among individuals. 

Here we used next-generation RNA-sequencing to produce a transcriptome-wide profile of the immune response of *A. cervicornis* to WBD by comparing infected versus healthy (asymptomatic) coral tissues. The transcriptome of *A. cervicornis* was assembled de novo from A-tail selected mRNA-seq data from whole coral tissues, and parsed bioinformatically into coral and non-coral transcripts using existing *Acropora* genomes in order to identify putative coral transcripts. Differentially expressed transcripts were identified in the coral and non-coral datasets to identify which genes were up- and down-regulated due to disease infection and characterize the immune response of the coral.

## Results

A *de novo* assembly of the *A. cervicornis* transcriptome was assembled from 436.5 million Illumina RNA-sequencing reads from 45 coral samples of *A. cervicornis* and *A. palmata*. The total reads were *de novo* assembled using *Trinity* [38], resulting in 95,389 transcripts, with a N50 of 363 and N75 of 696. A total of 47,748 transcripts mapped against the existing *Acropora* genomes [39,40] and were classified as putative coral transcripts while the remaining 47,641 were classified as non-coral transcripts (Table 1).

**Table 1:** Summary of coral and non-coral transcripts.

	*All Transcripts*						*Annotated (E-value < 10^-5^)*			
	*n*	*DE*	*%*	*Up*	*Down*		*n*	*DE*	*%*	*Up*
*Coral*	47,748	1805	3.78%	1460	345		20,502	559	3.70%	459
*Non-Coral*	47,641	551	1.16%	549	2		14,253	251	1.76%	251

Total number of transcripts (n), significantly differentially expressed transcripts (adj p-val<0.05) (DE), number of up-regulated (up) and down-regulated (down) transcripts among the entire dataset and annotated transcripts only (E-val<10^-5^). First row refers to putative coral transcripts, second row to non-coral transcripts.

For this study, five diseased (i.e. infected) and six healthy corals were used to profile the immune response of Staghorn corals infected with WBD. The average number of putative coral reads (±SE) was 4,076,829 (± 898,542) in the diseased coral samples compared to 4,199,946 (±761,894) in the healthy samples. In total, 20,503 coral transcripts (43 %) and 14,253 (30%) non-coral transcripts had strong protein annotations (Blastx e-value < 10^-5^) (Table 1).

### Differentially expressed coral transcripts

**Figure 2:**
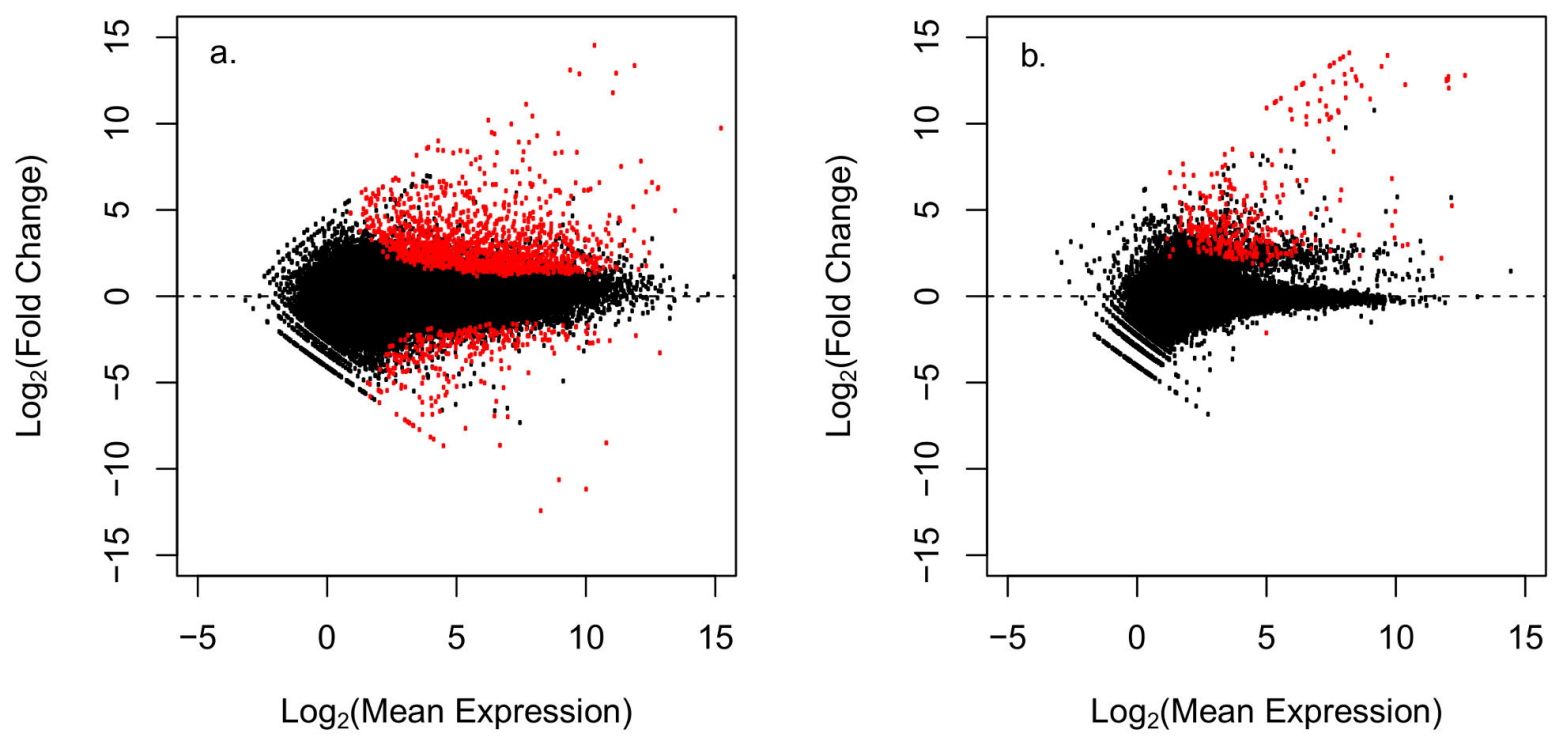
Volcano plots displaying differential gene expression between healthy and disease *A. cervicornis*. Figure a. plots gene expression values of the putative coral transcripts, figure b. plots putative non coral transcripts. Each point represents an individual gene transcript. Red points represent significantly differentially expressed transcripts (adj p-value < 0.05).

**Figure 3:**
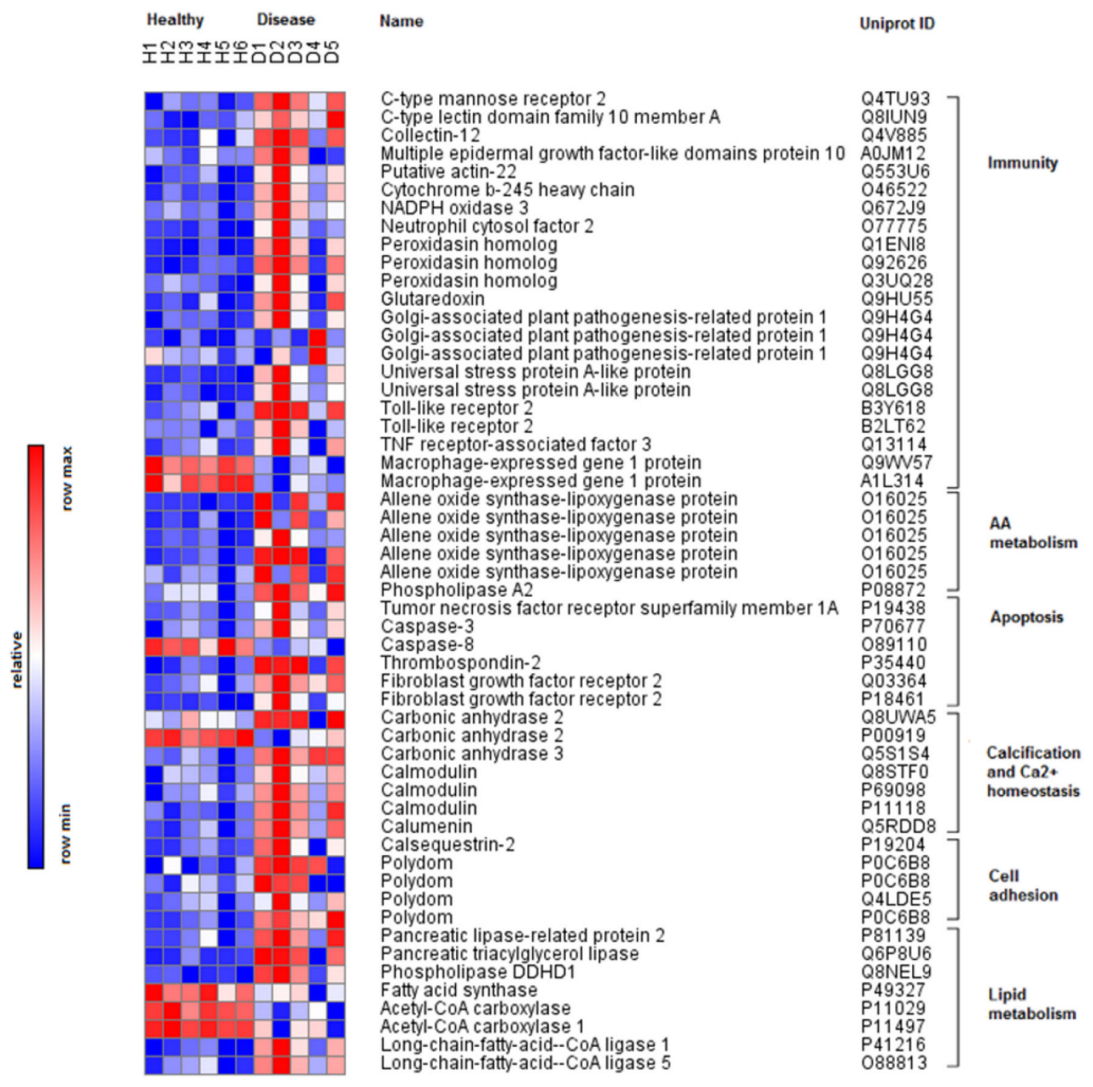
Heatmap of immune-related differentially expressed coral transcripts. Heatmap showing expression profiles of healthy (H) and WBD infected (D) *A.cervicornis*. Transcripts annotations are based on GO terms and manually curated categories. Relative expression levels are shown in red (up) and blue (down).

Statistical analysis in DEseq [41] identified 1,805 differentially expressed (DE) transcripts (adj p-value < 0.05) between healthy and WBD coral samples (Table 1, Table S1); 559 of these DE transcripts had reliable protein annotations (Blastx e-values < 10-5) that could be used to characterize the immune response of *A. cervicornis* infected with WBD (Figure 2a, Figure 3). Annotated transcripts were characterized by gene ontology (GO) and grouped into manually curated categories based on literature searches highlighting immune functions (Table 2). WBD-infected corals exhibited strong gene expression responses for genes related to immunity (n = 72), apoptosis (n = 18) and arachidonic acid metabolism (n = 5). Calcification (n = 14) and calcium homeostasis (n = 21) were also perturbed, as well as cell growth and remodeling (n = 134), cellular processes (n = 188) and general metabolism (n = 43). 

**Table 2:** Summary of the main pathways involved in *A.cervicornis* response to WBD.

*DE transcripts*	*N*	*Uniprot ID*	*Function*	*log_2_ (Fold Change)*
*Immunity*	69			
Macrophage-expressed gene 1 protein (n=2)		Q9WV57	complement related	2.2- 1.6 down
C-type lectin domain family 10 member A		Q8IUN9	pathogen recognition receptor	5.39 up
C-type mannose receptor 2		Q4TU93	pathogen recognition receptor	2.21 up
Collectin-12		Q4V885	pathogen recognition receptor	4.10 up
Multiple epidermal growth factor-like domains protein 10		A0JM12	phagocytosis	2.17 up
Putative actin-22		Q553U6	phagocytosis	1.56 up
Glutaredoxin		Q9HU55	response to oxidative stress	2.06 up
Peroxidasin homolog (n=3)		Q3UQ28	response to oxidative stress	3.1-6.6 up
Golgi-associated plant pathogenesis-related protein 1 (n=3)		Q9H4G4	response to stress	1.7- 3.5 up
Universal stress protein A-like protein (n=2)		Q8LGG8	response to stress	2.5- 2.6 up
Cytochrome b-245 heavy chain		O46522	superoxide anion generation	2.35 up
Neutrophil cytosol factor 2		O77775	superoxide anion generation	2.66 up
NADPH oxidase 3		Q672J9	superoxide anion generation	2.19 up
TNF receptor-associated factor 3		Q13114	toll	2.51 up
Toll-like receptor 2 (n=2)		B3Y618	toll	1.7-4.7 up
*Apoptosis*	18			
Caspase-3		P70677	apoptotic process	2.67 up
Caspase-8		O89110	apoptotic process	2.30 down
Tumor necrosis factor receptor superfamily member 1A		P19438	apoptotic process	1.83 up
Thrombospondin-2		P35440	pro-apoptosis	7.20 up
*Arachidonic acid metabolism*	6			
Phospholipase A2		P08872	arachidonic acid metabolism	3.03 up
Allene oxide synthase-lipoxygenase protein (n=5)		O16025	allene oxide synthesis	2.1 - 8.4 up
*Calcification*	14			
Carbonic anhydrase 2 (n=2)		Q8UWA5	one-carbon metabolic process	2.6 down, 2.7 up
Carbonic anhydrase 3		Q5S1S4	one-carbon metabolic process	1.77 up
*Calcium Homeostasis*				
Calmodulin (n=3)		Q8STF0	calcium ion binding	2.4-4.0
Calumenin		Q5RDD8	calcium ion binding	2.74 up
Calsequestrin-2		P19204	calcium ion binding	1.75 up
*Cell growth and remodeling*	135			
Polydom		P0C6B8	cell adhesion	1.9-5.3 up
Cellular processes	188			
*Metabolism*				
Fatty acid synthase		P49327	fatty acid biosynthetic process	2.28 down
Acetyl-CoA carboxylase		P11029	fatty acid biosynthetic process	1.88 down
Acetyl-CoA carboxylase 1		P11497	fatty acid biosynthetic process	2.08 down
Long-chain-fatty-acid--CoA ligase 1		P41216	fatty acid metabolic process	4.45 up
Long-chain-fatty-acid--CoA ligase 5		O88813	fatty acid metabolic process	4.35 up
Pancreatic triacylglycerol lipase		Q6P8U6	lipid catabolic process	4.23 up
Pancreatic lipase-related protein 2		P81139	lipid catabolic process	2.89 up
Phospholipase DDHD1		Q8NEL9	lipid catabolic process	2.28 up
*Wnt*	8			
*Unknown*	55			

Number (N) of differentially expressed (DE) transcripts per category. Function defined by GO terms and manually curated categories. Expression values reported as log_2_fold change of WBD infected corals relative to healthy corals.

### Immune-related processes

Sixty-nine DE transcripts were associated with immunity. Three C-type lectins receptors, C- type mannose receptor 2 (MRC2), macrophage lectin 2 (CLEC10A) and collectin-12 (COLEC12) were up-regulated in infected corals. Two mediators of phagocytosis were up-regulated - the macrophage receptor multiple epidermal growth factor-like domains protein 10 (MEGF10) and actin-22 (act22), which is involved in the phagosome formation. All three subunits of NADPH oxidase (NOX) involved in reactive oxygen species (ROS) production were up-regulated, including cytochrome b-245 heavy chain (CYBB), NADPH oxidase 3 (NOX3) and neutrophil cytosol factor 2 (p67-phox). Other DE immune related genes included nine antioxidants participating in the detoxification of ROS such as peroxidasin (PXDN, n=3) and glutaredoxin (GLRX), and 12 transcripts associated to response to stress such as golgi-associated plant pathogenesis-related protein 1 (GAPR-1, n = 3) and universal stress protein A-like protein (UspA, n = 2). 

Little or no differential expression was detected in the three primary innate immune pathways – Toll/TLR, complement and prophenoloxidase (PO) pathways. In the Toll/TLR pathway, two TLR2 homologs and the adaptor molecule TNF receptor-associated factor 3 (TRAF3) were up-regulated in WBD corals. In the complement pathway, two transcripts encoding macrophage-expressed gene protein 1 (MPEG1) were differentially expressed, but they were down regulated in WBD corals. No differentially expressed transcripts were detected in the PO pathway.

### Arachidonic acid metabolism

Six DE transcripts participating to the metabolism of arachidonic acid (AA) were up-regulated in diseased corals. Five matched coral allene oxide synthase-lipoxygenase (AOSL), a catalase related hemoprotein that catalyzes the biosynthesis of allene oxide, a precursor of marine eicoesanoids. The sixth transcript matched the enzyme phospholipase A2 (PLA2), involved formation of AA from membrane phospholipids.

### Apoptosis

Eighteen DE transcripts were associated with apoptosis, including both pro- and anti-apoptotic regulators such as the extracellular matrix protein thrombospondin 2 and fibroblast growth factor receptor 2 (n = 2), respectively. Tumor necrosis factor receptor superfamily member 1A (TNFRSF1A) and caspase 3 (CASP-3) were up-regulated while caspase 8 (CASP-8) was down-regulated in WBD corals. 

### Calcification and calcium homeostasis

DE transcripts in this category included 14 proteins participating to carbon dioxide transport, biomineralization and skeletal growth. Two carbonic anhydrases were up-regulated (CA2 and CA3) and one was down-regulated (CA2) in WBD corals. Mediators of calcium homeostasis included 27 DE transcripts participating in calcium ion binding and transport such as calmodulin (CaM, n = 3), calumenin (CALU) and calsequestrin-2 (CASQ2) and were all up-regulated.

### Cell growth and remodeling

Among the 138 DE transcripts related to cell growth and remodeling we identified 17 metallopeptidases (15 up, 2 down), 29 cytoskeletal proteins (all up-regulated) and 14 angiogenesis mediators (11 up, 3 down). A large group of DE transcripts were cell adhesion proteins (n = 29), including four up-regulated transcripts encoding sushi, von Willebrand factor type A, EGF and pentraxin domain-containing protein 1 (polydom/SVEP1).

### Cell metabolism

Forty-two DE transcripts were associated with cell metabolism. These included 14 mediators of lipid metabolism, in particular, five lipases involved in lipid and phospholipid catabolism (n = 5, all up), such as pancreatic triacylglycerol lipase (PL), pancreatic lipase-related protein 2 (PL-RP2) and phospholipase DDHD1 (DDHD1). Four transcripts participating in fatty acid biosynthesis, such as fatty acid synthase (FASN), acetyl-CoA carboxylase (ACC) and acetyl-CoA carboxylase 1 (ACC1), were all down-regulated in WBD corals, and five transcripts involved in the breakdown of fatty acids such as long-chain-fatty-acid--CoA ligase 1 (LACS1) and 5 (LACS5) were up-regulated.

### Non-coral transcripts

Out of the 47,641 putative non-coral transcripts in the dataset, 550 were differentially expressed in WBD infected corals (Table 1, Table S2). Of these 550 DE transcripts, 251 were well-annotated and were all up-regulated (Figure 2b). About 33 % were metazoan, the remaining were putative zooxanthellae (23%), fungi (10%) and protozoa (5%). A small number of transcripts matched bacteria (4%) and viruses (0.1%), while the remaining 23 % were unknown.

Metazoan transcripts (n = 84) included mediators of cell growth and remodeling (n = 16), metabolism (n = 4), cellular processes (n = 61) and two uncharacterized transcripts. Only two immune-related transcripts were identified and were the antioxidant peroxiredoxin-2 (PRDX2) and the metallopeptidase aminopeptidase O (AP-O), which may be involved in leukotrienes synthesis from AA. 

Fifty-nine transcripts had plant, algae or Alveolata protein IDs and are presumed or putative *Symbiodinum* transcripts. Based on GO terms, these *Symbiodinium* transcripts were associated with cell growth and remodeling (n = 8), cellular processes (n= 38) and metabolism (n = 10), while two were uncharacterized. One transcript matched cysteine proteinase RD21a (RD21), a peptidase involved in defense against fungi. Fungal transcripts (n = 24) belonged to cell processes (n = 20) and metabolism (n = 3) plus one uncharacterized protein. Out of the 14 transcripts matching protozoa, 13 were associated to cellular processes, two to metabolism and one to cell growth and remodeling. 

Nine transcripts matched bacterial proteins, six of them were involved in cellular processes (n = 3), metabolism (n = 3) and three were uncharacterized. Two transcripts shared protein IDs annotating to virus proteins (glycoprotein gp2 and one uncharacterized), while the remaining 59 transcripts did not have functional annotations.

## Discussion

Our study demonstrates that *Acropora cervicornis* mounts a vigorous immune response against White Band Disease (WBD) pathogen(s) involving dramatic changes in gene expression across 4% of the coral transcriptome. The identities of the differentially expressed (DE) coral transcripts indicate that the response of *A. cervicornis* to WBD infection is driven by phagocytosis of apoptotic cells (Figure 3, Table 2). Corals infected with WBD exhibited strong differential expression of transcripts involved in macrophage-mediated pathogen recognition and ROS production, two hallmarks of phagocytosis, as well as key mediators of apoptosis and calcium homeostasis. The strong up-regulation of transcripts involved in arachidonic acid (AA) metabolism and allene oxide synthesis suggests their key role in coral immunity. 

The primary signature of phagocytosis activity in WBD infected corals was the up-regulation of four macrophage receptors that recognize and bind to conserved motifs on the surface of target cells. Three of these receptors, MRC2, CLEC10A and COLEC12 belong to the C-type lectin family of proteins that include several Pathogen Recognition Receptors (PRRs). MRC2 recognizes mannose and fucose on glycoproteins of bacteria, viruses and fungi [42] while CLEC10A recognizes galactose and N-acetyl-galactosamine residues [43]. COLEC12is a scavenger receptor that shares structural similarity with macrophage scavenger receptor class A type I (SR-AI), a surface membrane receptor that mediates binding and phagocytosis of gram-positive, gram-negative bacteria and yeasts [44]. The fourth receptor, MEGF10, is membrane protein that promotes the clearance of apoptotic cells by causing macrophages to adhere and engulf them [45]. The stronger up-regulation of the three macrophage PRRs (2.2, 5.4 and 4.1 fold) compared to the one apoptotic cell recognizing receptor MEGF10 (2.17 fold) suggests the response is primarily driven by phagocytosis of microbes. A second signature of phagocytosis was the up-regulation of transcripts linked to ROS production, including three subunits of the enzymatic complex NADPH oxidase (NOX). ROS production is a general and highly conserved response to invading pathogens and stress and the release of ROS from the mitochondria can induce apoptosis in metazoan and yeasts [46,47]. During phagocytosis, ROS are generated in mature phagosomes (i.e. specialized vacuoles in phagocytic cells) [48] to kill engulfed cells [49]. In cnidarians, ROS production has been observed in the hydroid *Hydra vulgaris* exposed to the immune stimulant lipopolysaccaride (LPS) [50] and in reef corals during thermal and UV-induced bleaching [51,52], possibly due to the breakdown of the mitochondrial and photosynthetic membranes [53,54]. 

In WBD infected corals, it is possible that phagocytosis is aimed either at the removal of invading pathogens and/or used to clear damaged apoptotic cells [55]. The genetic signature of phagocytosis in WBD infected corals raises questions about the identity of these phagocytic immune cells in *A. cervicornis*. Cnidaria lack specialized immune cells, but do possess mobile amebocytes. Aggregations of amebocytes have been observed in the gorgonian coral *Gorgonia ventalina* infected with pathogenic fungi [24] and near wounded tissues in the soft coral *Plexaurella fusifera* [26]. Histological examination revealed that amebocytes exhibited phagocytic and PO activity [27] as well as antimicrobial activity against Gram-negative bacteria and ROS production [56]. Interestingly, certain populations of ameboid cells always show phagocytic activity, while others only acquire it upon immune activation [27]. These ﬁndings indicate that cnidaria, traditionally considered “simple” animals, are able to mount an innate immune response by employing the functional plasticity of amebocytes, which seem to represent the primary immune population of phagocytic cells.

Increased apoptosis in WBD infected corals was indicated by the differential expression of TNFRSF1A and CASP-3. During apoptosis, TNFRSF1A binds to tumor necrosis factor (TNF), which then recruits CASP-8 initiating the downstream activation of CASP-3, the main effector caspase of the apoptotic pathway [57,58]. While both TNFRSF1A and CASP-3 are up-regulated, CASP-8 is down-regulated which may suggest that CASP-3 is activated by some alternative pathway. Active programmed cell death was also suggested by disruption of calcium homeostasis as indicated by the strong up-regulation of CaM and other calcium binding proteins. In both plants and animals [59,60], apoptosis can be triggered by LPS from gram-negative bacteria via alteration of TNFRSF1A expression [61]. Some bacterial pathogens are also able to induce or inhibit apoptosis in their host [60,62,63] via alteration of membrane permeability and disruption of Ca2+ homeostasis [64], direct activation of TNF-α [65], TLR2 [66,67]or CASP-3 [68]. In corals, apoptosis occurs normally during metamorphosis [69] and the onset of symbiosis [70], but it has also been observed during bleaching as a possible mechanism to expel zooxanthellae in response to thermal stress [71-73]. Apoptosis has also been detected in the lesions of three Pacific species of *Acropora* infected by White Syndrome (WS), suggesting that it is a mechanism of tissue loss in WS [74]. 

Another key, yet unexpected, finding of this study is the potential role of the arachidonic acid (AA) pathway in the coral immune response. Genes involved in AA synthesis increased dramatically in WBD infected corals. The role of AA as an inflammation regulator is well-known in metazoans [75] , but has not been described in Cnidaria or in association with any coral disease. In metazoans, AA is released by apoptotic cells as chemotactic factor to promote clearance by phagocytes [76], but it can also induce apoptosis via rapid increase of calcium concentration and activation of CASP-3 in a CASP-8-independent way [77]. These findings are consistent with our data showing up-regulation of CASP-3, but not CASP-8, suggesting that AA may act similarly as immunomodulator in *A. cervicornis*. The five transcripts matching allene oxide synthase-lypoxigenases (AOSL) from the soft coral *Plexaura homomalla*, on the other hand, indicated that AA is converted into allene oxide, an intermediate compound of prostanoid synthesis in plants and soft corals [78-82].

Allene oxide has received considerable attention as a putative precursor of clavulones [83], a class of unique marine prostanoids known for their anti-viral and anti-cancer activity [84,85]. The link between the AOSL pathway and clavulones synthesis in corals, although still under debate, was suggested by the similarities with the biosynthetic pathway of jasmonic acid [83] a plant hormone that is produced via an allene oxide intermediate upon mechanical injury [86] and herbivore attack [87]. Although further study is needed to understand the role allene oxide in corals, our data represent the first evidence implicating AOSL in coral immunity and suggest that AOSL may be involved in controlling levels of free AA produced by apoptotic cells. 

Several other immune related genes exhibited altered expression in infected corals. The majority were anti-oxidants including PXDN, peroxidasin-like proteins and GLRX - a glutathione-dependent enzyme. PXDN has been shown to be DE in some thermally stressed corals, but not in a consistent manner. For example, in *Montastraea faveolata*, PXDN was up-regulated in thermally-stressed larvae [88], but was down-regulated in thermally-bleached adult colonies [89]. Active cell remodeling and cell matrix degradation was indicated by several DE cytoskeletal proteins, metalloproteases and cell adhesion proteins, probably associated with cellular and cytoskeletal rearrangements linked to phagocytosis and apoptosis. CASP-3 activation, in particular, initiates apoptosis by altering the expression of metalloproteases and hydrolytic enzymes such as cathepsins that degrade extracellular matrix components [90]. Interestingly, WBD infected corals up-regulated three transcripts encoding polydom, a cell adhesion protein belonging to the pentraxin family of lectins. Recent studies suggest an immune function for polydom based on its similarities in its protein domains to complement proteins and C-type lectins with antimicrobial activity [91]. In cnidarians, the potential immune role for polydom is bolstered by its up-regulation in the hydroid *Hydractinia symbiolongicarpus* after fungal and bacterial exposure [92].

Surprisingly, none of the three main innate immune pathways - TLR, complement and PO - played a prominent role in the immune signature of *A. cervicornis* infected with WBD, even though transcripts from these pathways are well-represented in our transcriptome. Only three transcripts in the TLR pathway were differentially expressed: two TLRs matching to human TLR2 and TRAF3. In the lectin complement pathway, the only two DE transcripts were two proteins matching MPEG1, a MAC/PF (membrane attack complex/perforin) containing protein that is involved in the response against Gram negative bacteria in sponges and is up-regulated upon LPS exposure [93]. None of the transcripts belonging to the PO pathway were differentially expressed during WBD infection, even though in other corals PO activity acts as an important defense against invading pathogens and tissue damage [16-18].

### Non-coral transcripts

The taxonomic distribution of non-coral transcripts highlighted the presence of several members of the coral holobiont, i.e. the coral host and associated symbiotic microorganisms, including zooxanthellae, fungi and protozoa. The majority of these non-coral transcripts matched metazoan and putative zooxanthaellae proteins, while the remaining transcripts matched fungi, protozoa and bacteria. GO term analysis revealed that most of these non-coral transcripts encoded mediators of cell homeostasis and general metabolism. Transcripts with metazoan identities were likely coral transcripts that did not have identities in the coral reference genomes and may thus represent transcripts unique to *A. cervicornis*. Putative zooxanthellae transcripts were identified as transcripts annotating to Viridiplantae, Heterokontophyta (i.e. algae), cyanobacteria and the superphylum Alveolata. Interestingly, no genetic signature of immune activity from the algal symbionts was evident in our transcriptome. Instead, our data suggest drastic changes in photosynthesis and cell metabolism of the zooxanthellae; this is consistent with a previous study showing that *Symbiodinum* undergo major alteration of carbon metabolism in response to stress [94]. 

## Conclusions

Our data reveal that the coral host, but not its algal symbionts, undergoes dramatic alterations in gene expression during response to WBD infection. Transcriptional changes affected mediators of innate immunity, in particular receptors on the surface of phagocytic cells, enzymes involved in ROS production and modulators of apoptosis. Taken together, our data suggest that WBD infection in *A. cervicornis* is associated with apoptosis, and that WBD pathogen triggers a powerful immune response driven by phagocytic cells that encapsulate and degrade apoptotic cells. This study also indicates a key role for arachidonic acid and in particular the enzyme AOSL in *A. cervicornis* immunity. 

## Materials and Methods

Total RNA was extracted from diseased and healthy *Acropora cervicornis* sampled from Crawl Cay reef in Bocas del Toro, Panama under Autoridad Nacional del Ambiente (ANAM) Collecting permit SE/A-71-08. For the diseased samples, corals with active mobile WBD interfaces were identified by monitoring the mobility of disease interfaces for two days, and then sampling a 2 cm region of tissue at and above the disease interface. A comparably sized and located tissue sample was taken from healthy (i.e. asymptomatic) corals. The coral tissues were flash frozen in liquid nitrogen and stored at -80°C. Total RNA was extracted in TriReagent (Molecular Research Center, Inc.) following the manufacturer's protocol. Total RNA quality was assessed using the RNA Pico Chips on an Agilent Bioanalyzer 2100, and only extractions showing distinctive 28S and 18S bands and RIN values of 6 or higher were prepped for RNA sequencing.

RNA sequencing was performed on five diseased and six healthy coral samples using a multiplexed Illumina mRNA-seq protocol [95] with the following modifications. Instead of fragmenting the mRNA prior to cDNA synthesis, we obtained much better success fragmenting the double stranded cDNA using DNA fragmentase (New England Biolabs) for 30 minutes at 37°C. RNA-seq libraries were then prepared using next-generation sequencing modules (New England Biolabs) and custom paired-end adapters with 4bp barcodes. Multiplexed samples were run (2-3 samples per lane) on the Illumina GAII platform (Illumina, Inc, San Diego, California, USA) at the FAS Center for System Biology at Harvard University. Barcoded samples were de-multiplexed and raw sequencing reads were quality trimmed to remove sequences and regions with a Phred score of less than 30 and a read length less than 15bp long using custom Perl Scripts in the FASTX-Toolkit (http://hannonlab. cshl.edu/fastx_toolkit/).

A *de novo* transcriptome was assembled using *Trinity* [38] from 463.5 million single-end Illumina RNA-Seq reads from 39 *A. cervicornis* and 6 *A. palmata* samples, including the 11 *A. cervicornis* samples included in this paper. The assembled transcriptome produced 95,389 transcripts with a N50 of 363 and N75 of 696. RNA-seq data were produced using whole coral tissue, which putatively contains sequences from the coral host, its algal symbiont *Symbiodinium*, and other members of the coral holobiont (e.g. fungi, bacteria, and viruses).

In order to resolve the holobiont, and putatively classify the source of the transcripts that were assembled as either *coral* or *non-coral*, we utilized a multistep pipeline leveraging the existing genomes of two congener species – *A. digitifera* [39] and *A. millepora* [40]. RNA-seq reads were mapped against both *Acropora* reference genomes using Bowtie [96] to produce two exomes. Transcripts from our *de novo* assembly were aligned using BLAST [97] against each exome. Transcripts were assigned as putatively coral if they matched either exome with an e-value of less than 10^-10^. Transcripts without significant coral hits were assigned as non-coral and could potentially include novel coral and/or algal symbiont *Symbiodinium* transcripts, as well as other associated eukaryotes, like endolithic fungi. Bacterial and viral transcripts are possible, but less likely given that A-tail selection to isolate eukaryotic mRNAs was performed prior to cDNA synthesis.

Putative gene identities for each transcript were identified by performing homology searches against the Swiss-Prot and TReMBLE protein databases [98], using tBLASTx. Matches with an e-value of less than10^-5^ were considered homologous protein-coding genes. Subsequently, GenBank Flat Files corresponding to the hits’ Accession ID’s were downloaded and used to extract taxonomic data for each used as a second method to identify the putative source of the transcripts. GO terms and gene functions were obtained for the annotated transcripts on UniProt. The reference transcriptome sequences are available on Bioproject (accession number PRJNA222758).

Differences in gene expression between healthy and disease *A.cervicornis* specimens were estimated using the R package DESeq [38]. First, all contigs were separated into two datasets –i.e. coral and non-coral- based on their matches to the *Acropora* genomes. Size factor estimation and normalization were then performed separately on each dataset using the functions estimateSizeFactors and estimateDispersions, respectively. Differentially expressed contigs were detected by running a negative binomial test using the function nbinomTest. Only differentially expressed transcripts (adjusted p-value < 0.05) that were also annotated (e-values < 10^-5^) were used for this study.

## Supporting Information

Table S1 **Dataset of annotated (E-val<10^-5^ ) coral transcripts exhibiting differential expression between healthy and diseased samples (adj p-val<0.05).**
(XLSX)

Table S2 **Dataset of annotated (E-val<10-5 ) non-coral transcripts exhibiting differential expression between healthy and diseased samples (adj p-val<0.05).**
(XLSX)
